# A new method for evaluating tumor-infiltrating lymphocytes (TILs) in colorectal cancer using hematoxylin and eosin (H-E)-stained tumor sections

**DOI:** 10.1371/journal.pone.0192744

**Published:** 2018-04-26

**Authors:** Yasuhito Iseki, Masatsune Shibutani, Kiyoshi Maeda, Hisashi Nagahara, Tatsunari Fukuoka, Shinji Matsutani, Shinichiro Kashiwagi, Hiroaki Tanaka, Kosei Hirakawa, Masaichi Ohira

**Affiliations:** Department of Surgical Oncology, Osaka City University Graduate School of Medicine, Abeno-ku, Osaka, Japan; National Cancer Center, JAPAN

## Abstract

**Purpose:**

Numerous reports indicate that tumor-infiltrating lymphocytes (TILs) are a prognostic factor in various cancers and that they must be good biomarkers. However, the methods of evaluating TILs differ in each study; thus, there is not yet a standardized methodology for evaluating TILs. The purpose of this study is to evaluate the prognostic significance of tumor-infiltrating lymphocytes (TILs) in patients with colorectal cancer (CRC) using the new method proposed by the International TILs Working Group in breast cancer and to standardize the method of evaluating TILs in CRC.

**Methods:**

We retrospectively reviewed a database of 160 patients with Stage II or III CRC. The density of TILs was assessed by measuring the area occupied by mononuclear cells over the stromal area on hematoxylin and eosin (H-E)-stained sections. We set 42% as the cut-off percentage of the area occupied by TILs according to the receiver operating characteristic curve, and we classified patients into the high-TILs and the low-TILs groups.

**Results:**

The rates of relapse-free survival (RFS) and overall survival (OS) in the high-TILs group were significantly higher than those in the low-TILs group. A multivariate analysis showed that the density of TILs was independently associated with RFS and OS. Moreover, the density of TILs assessed by an observer was significantly associated with the density of TILs assessed by the automated imaging software program.

**Conclusions:**

The new method for evaluating TILs, which was recommended by the International TILs Working Group in breast cancer, might be a useful predictive factor in colorectal cancer patients.

## Introduction

Colorectal cancer (CRC) is the third most common cancer in the world [1]. Although the surgical approach and chemotherapy for CRC have improved, the prognosis remains poor, as one-third of patients who undergo curative resection die within five years after surgery [2]. Thus, it is necessary to provide individualized therapy according to the risk stratification and to find biomarkers that can predict the prognosis in order to improve the prognosis. In addition to tumor factors, the local tumor environment (*i*.*e*., extracellular matrix, immune cells, and cytokines) has an important role in tumor growth, invasion, metastasis and proliferation. Thus, these are considered to be prognostic factors in patients with colorectal cancer [3–8].

In particular, tumor-infiltrating lymphocytes (TILs) are associated with the immune status of host and various reports have shown the TIL level to be a favorable biomarker in the prognosis of numerous cancers, including colorectal cancer [3–6]. However, the methods of evaluating TILs differ in each study; thus, there is not yet a standardized methodology for evaluating TILs.

In 2014, the International TILs Working Group recommended the standardization of the approach to measuring the density of TILs in breast cancer [9]. They showed that it is possible to evaluate the density of TILs on H-E-stained sections without evaluating the subsets of lymphocytes.

The aim of the present study is to assess the prognostic utility of TILs on H-E-stained sections in colorectal cancer in the same way as breast cancer and to standardize the methodology for evaluating TILs.

## Patients and methods

### Patients

We retrospectively reviewed a database of 160 patients who underwent curative surgery for Stage II/III CRC at the Department of Surgical Oncology, Osaka City University, Japan between January 2007 and November 2009. Patients who had a history of cancer therapy, who had undergone neoadjuvant chemo/chemoradiotherapy, who had undergone emergency surgery for various symptoms caused by cancer, or who had a history of inflammatory bowel disease were excluded from the present study. Written informed consent for participation in the present study was obtained from the patients. This study was performed in compliance with the principles expressed in the Declaration of Helsinki, and was approved by the ethics committee of Osaka City University (approved no. 3853). The resected specimens were judged according to the International Union Against Cancer (UICC) staging classification of colorectal cancer [10]. All patients were followed up until death or December 2015.

The patients with Stage III or high-risk Stage II disease were included in the indications for adjuvant chemotherapy. High-risk Stage II disease was defined by T4 tumors, lymphatic vessel invasion, blood vessel invasion and/or high-grade histology. In some of these patients, we didn’t administer adjuvant chemotherapy because of their performance status, general condition, age and/or their wishes. One hundred three patients (64.8%) received adjuvant chemotherapy. Patients were given an oral or intravenous regimen that included fluorouracil.

Regarding relapse free survival (RFS), relapse was defined as cancer recurrence; death from any cause was also treated as relapse. The RFS was determined from the date of operation to the date of relapse, the date of being lost to follow-up, the date of death from any cause, or December 31, 2015, whichever occurred first.

Death from any cause was defined as the determination of the overall survival (OS). The OS was determined from the date of operation to the date on which the patient was lost to follow-up, the date of death, or December 31, 2015, whichever occurred first.

### Methods

**Fig 1:**
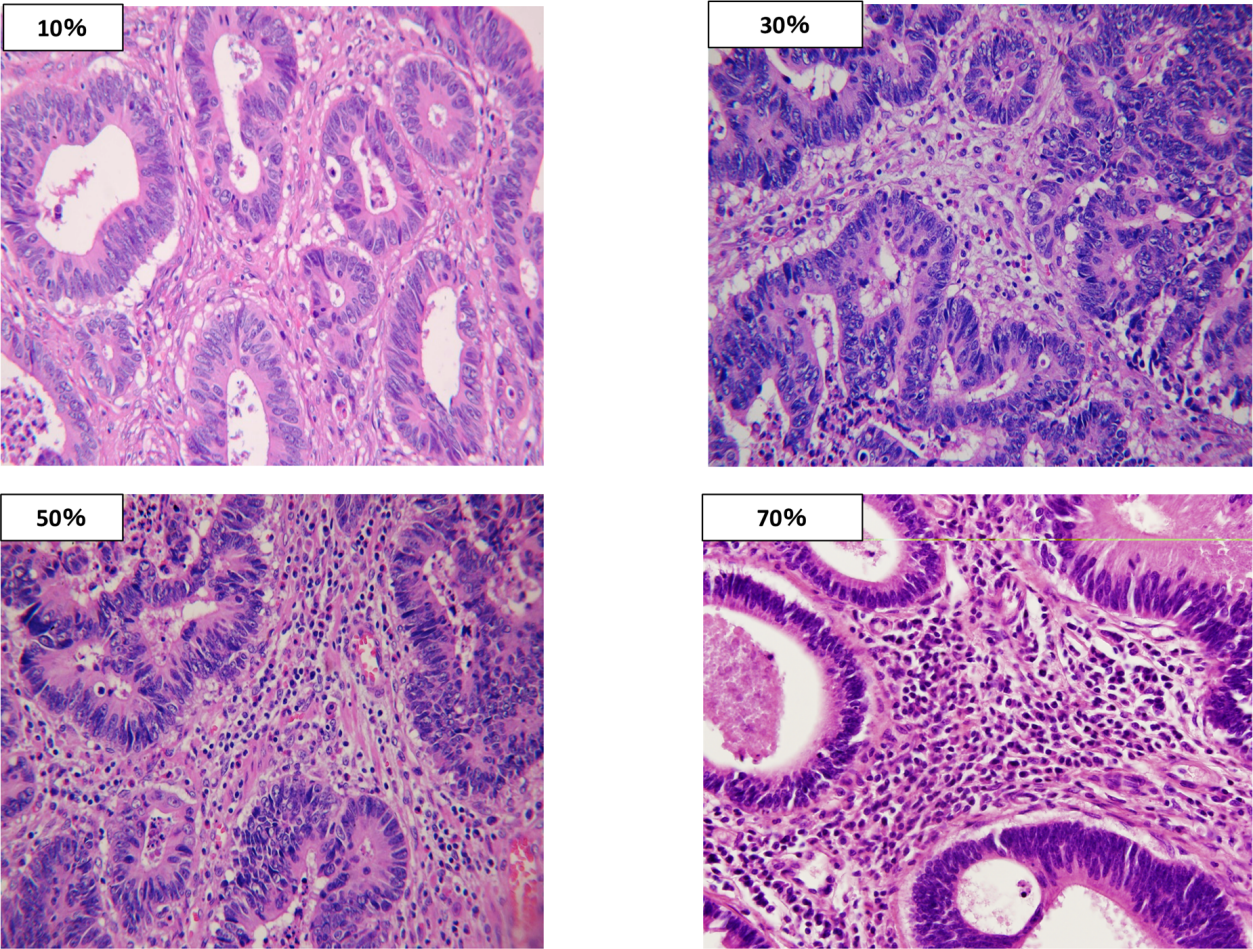
Examples of the evaluation of tumor infiltrating lymphocytes. Examples of the evaluation of tumor infiltrating lymphocytes(TILs) in the present study. The mean percentage of the area occupied by TILs in 5 areas per section was reported as the density of TILs.

We performed curative resection for colorectal cancer and estimated the density of TILs on hematoxylin and eosin (H-E) stained sections. The density of TILs was determined based on the recommendation by the International TILs Working Group [9]. We selected the tumor area at low magnification and assessed the percentage of the area that was filled with mononuclear cells in the stromal area around the tumor border at high magnification (×200). We defined all of the mononuclear cells, including the lymphocytes in the stromal area, as TILs and excluded granulocytes and other polymorphonuclear leukocytes. We used the recommendation of the International TILs Working Group as reference for evaluating the density of TILs. Examples of the evaluation are shown in Fig 1.

### The image analysis using an automated software program

**Fig 2:**
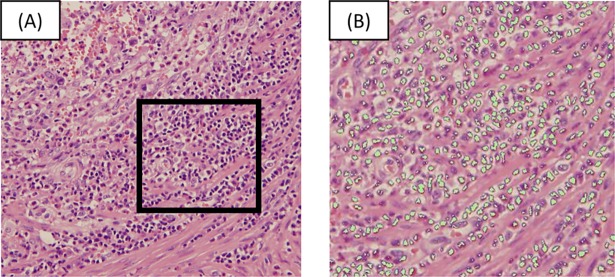
The method of identifying the area of TILs by the automated imaging software program. **(A)** A hematoxylin and eosin-stained section (×200). (B) Examples of the evaluating the area of TILs using the automated imaging software program (×400). The area in the square in Figure (A) was subjected to an image analysis and is shown in Figure (B). We set the depth of color of TILs. The cells of the same color were then extracted. The ratio of the white area to the extracted area was determined.

Micro Analyzer^®^ (Ver1.1d.1.1.54. Japan Pola Digital Company, Tokyo, Japan), an automated imaging software program, was used to evaluate the percentage of the area occupied by TILs. In the same way as assessing by an observer, the density of TILs was assessed by measuring the area occupied by mononuclear cells over the stromal area (Fig 2). The mean percentage of the area occupied by TILs in 5 areas per section was reported as the density of TILs.

### Statistical analysis

The JMP 11 software program (SAS Institute, Cary, NC, USA) was used to analyze the data. Differences between groups were analyzed using the χ^2^ test and the Wilcoxon signed rank test. The duration of survival was calculated according to the Kaplan-Meier method and differences in the survival curves were assessed using the log-rank test. The correlation between two variables was assessed using Spearman’s test. A multivariate analysis of the clinicopathological factors correlated with survival was performed using a Cox proportional hazards model. P values of <0.05 were considered to indicate statistical significance. Factors with a P value of < 0.1 on the univariate analyses were included in the multivariate analysis.

## Results

### Patient characteristics

The characteristics of 160 CRC patients are shown in Table 1.

**Table 1:** The patient characteristics.

Sex (male *vs*. female)	84 (52.5%) *vs*. 76 (47.5%)
Age (years) median(range)	67 (26–90)
Tumor size (cm) median(range)	4.7 (1.0–11.0)
Tumor location (colon *vs*. rectum)	86 (53.8%) *vs*. 74 (46.3%)
Preoperative CEA level (ng/ml) median(range)	3.5 (0.8–349.3)
Preoperative CA19-9 level (U/ml) median(range)	8.0 (1.0–1979)
Histology (low grade *vs*. high grade)	147 (91.9%) *vs*. 13 (8.1%)
Depth of tumor invasion (T1, 2, 3 *vs*. T4)	106 (66.3%) *vs*. 54 (33.8%)
Lymphatic vessel invasion (negative *vs*. positive)	38 (23.8%) *vs*. 122 (76.3%)
Venous invasion (negative *vs*. positive)	128 (80.6%) *vs*. 31 (19.4%)
Lymph node metastasis (negative *vs*. positive)	90 (56.3%) *vs*. 70 (43.8%)
Adjuvant chemotherapy (negative *vs*. positive)	56 (35.2%) *vs*. 103 (64.8%)
Mismatch repair status (proficient *vs*. deficient)	147 (94.2%) *vs*. 9 (5.8%).

CEA, carcinoembryonic antigen; CA19-9, carbohydrate antigen 19–9.

The study population included 84 males and 76 females. The median follow-up time was 63.5 months. Eighty-six patients had colon cancer and 74 patients had rectal cancer. 147 patients had low-grade tumors, and 13 had high-grade tumors. One hundred twenty-two patients had lymphatic vessel invasion, 31 had venous invasion, 70 had lymphocyte metastasis and 9 had mismatch repair deficiency.

**Fig 3:**
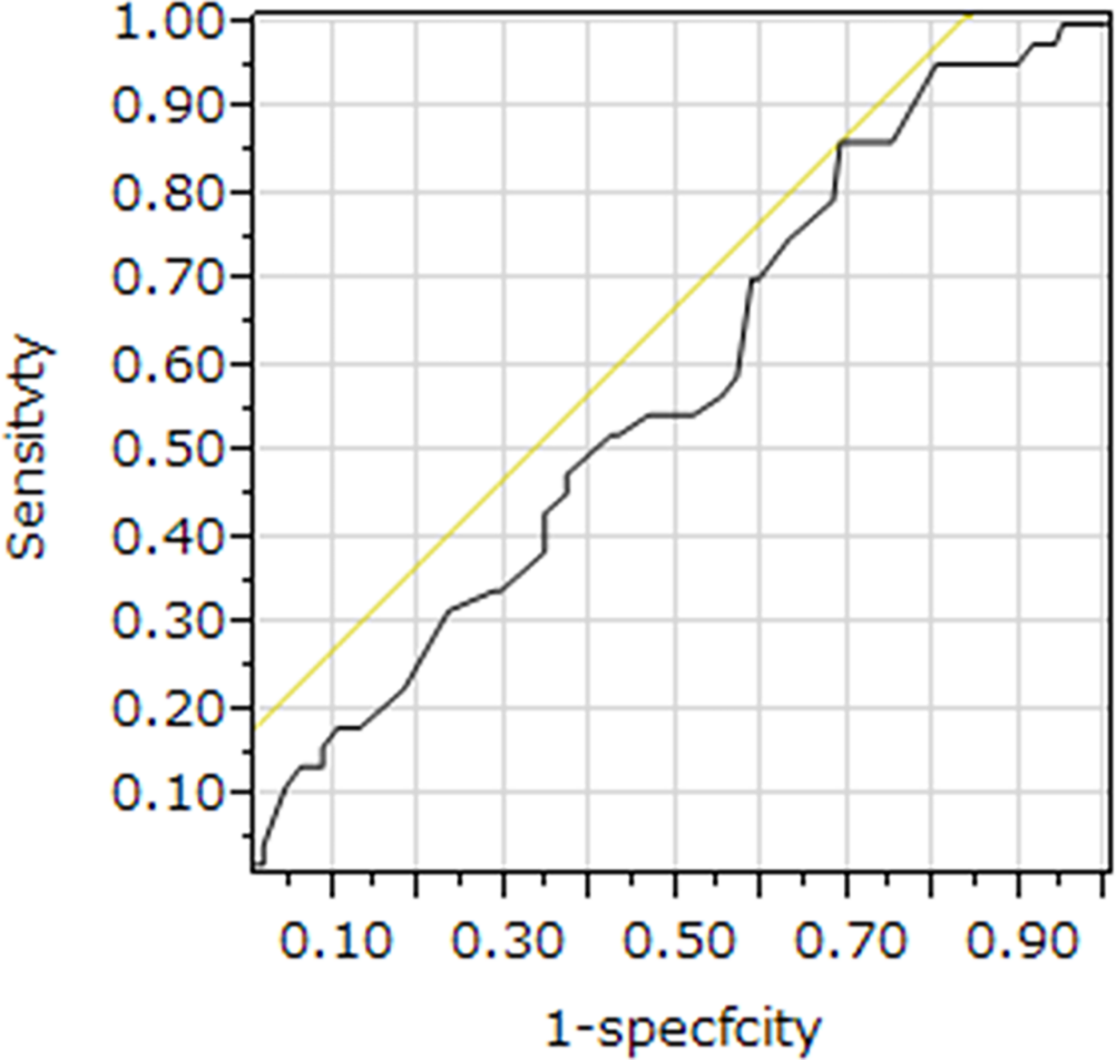
The receiver operating characteristic (ROC) curve for the density of tumor infiltrating lymphocytes (TILs). We treated the five-year relapse-free survival as the state variable and the percentage of tumor infiltrating lymphocytes (TILs) as the test variable. The most appropriate cut-off value for the percentage of TILs to be 42% was shown by the investigation of the cut-off value of percentage of TILs using the receiver operating characteristic (ROC) curve (AUC, 0.5781; sensitivity, 0.8636; specificity, 0.3103).

We treated the five-year relapse-free survival as the state variable and the percentage of tumor infiltrating lymphocytes (TILs) as the test variable. The most appropriate cut-off value for the percentage of TILs to be 42% was shown by the investigation of the cut-off value of percentage of TILs using the receiver operating characteristic (ROC) curve (AUC, 0.5781; sensitivity, 0.8636; specificity, 0.3103). The ROC curve is showed on Fig 3.

Accordingly, we decided 42% as the cut-off value for the percentage of TILs in this study and classified the patients into high TILs group (≧42%) and low TILs group (<42%).

The correlations between the patient characteristics and the density of TILs are shown in Table 2.

**Table 2:** The correlations between the clinicopathological factors and the area of tumor infiltrating lymphocytes.

	The density of TILs		
	High (N = 42)	Low (N = 118)	p-value
Sex			
Male	19 (11.9%)	65 (40.6%)	0.2726
Female	23 (14.4%)	53 (33.1%)	
Age (years)			
Median(range)	68 (34–89)	65 (26–90)	0.1594
Tumor location			
Colon	22 (13.8%)	64 (40.0%)	0.8359
Rectum	20 (12.5%)	54 (33.8%)	
Tumor size (cm)			
Median(range)	5.1 (1.0–10.0)	4.5 (1.0–11.0)	0.1874
Depth of tumor invasion			
T1,2,3	33 (20.6%)	73 (45.6%)	0.0435
T4	9 (5.6%)	45 (28.1%)	
Lymph node metastasis			
Negative	27 (16.9%)	63 (39.4%)	0.2187
Positive	15 (9.4%)	55 (34.4%)	
Lymphatic vessel invasion			
Negative	15 (9.4%)	23 (14.4%)	0.0391
Positive	27 (16.9%)	95 (59.4%)	
Venous invasion			
Negative	38 (23.9%)	90 (56.6%)	0.0135
Positive	3 (1.9%)	28 (17.6%)	
Preoperative CEA level			
≤5 ng/ml	30 (18.8%)	73 (45.6%)	0.2609
>5 ng/ml	12 (7.5%)	45 (28.1%)	
Preoperative CA19-9 level			
≤ 37 U/ml	39 (24.8%)	99 (63.1%)	0.2273
>37 U/ml	3 (1.9%)	16 (10.2%)	
Histology			
Moderately or Well	40 (25.0%)	107 (66.9%)	0.3282
Other	2 (1.3%)	11 (6.9%)	
Adjuvant chemotherapy			
Negative	15 (6.3%)	41 (25.8%)	0.8321
Positive	26 (16.4%)	77 (48.4%)	
Mismatch repair status			
Proficient	40 (25.6%)	107 (68.6%)	0.7385
Deficient	2 (1.3%)	7 (4.5%)	

TILs, tumor infiltrating lymphocyte; CEA, carcinoembryonic antigen; CA19-9, carbohydrate antigen 19–9.

The tumor depth (p = 0.0435), lymphatic vessel invasion (p = 0.0391) and venous invasion (p = 0.0135) were correlated with the density of TILs.

### Survival curves

**Fig 4:**
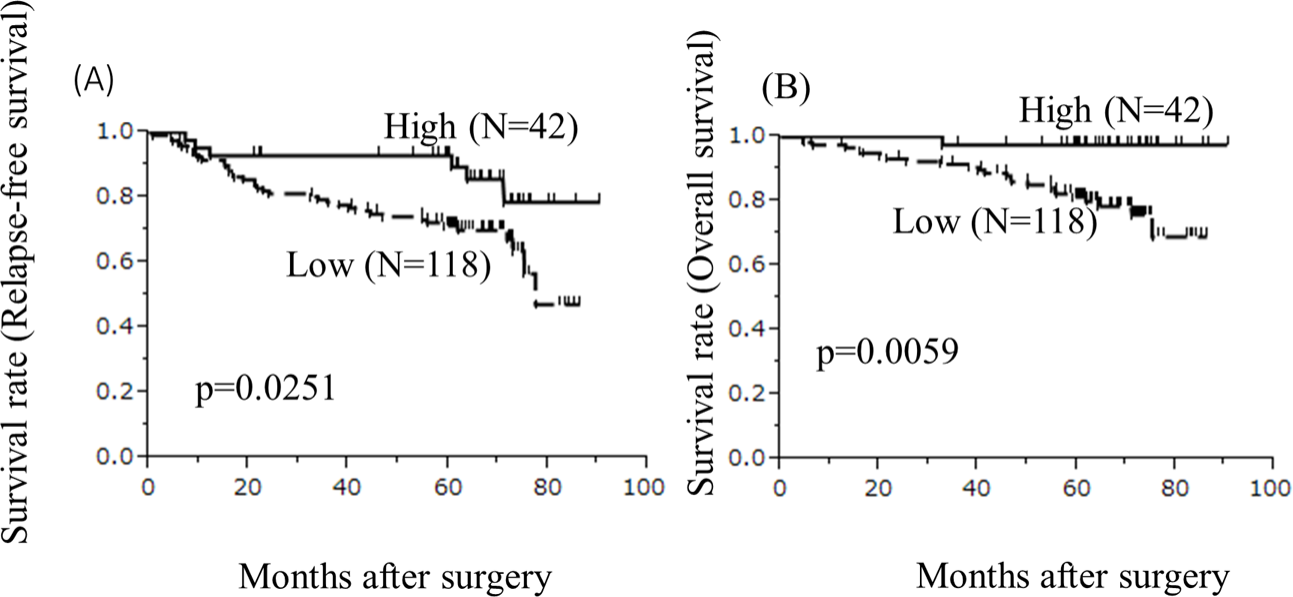
Survival curves. **(A)**The survival curves for relapse-free survival. The relapse-free survival rates of the low-TIL group were significantly worse in comparison to the high-TIL group (p = 0.0251). (B)The survival curves for overall survival. The overall survival rates in the low-TIL group were significantly worse in comparison to the high-TIL group (p = 0.0059).

The five-year relapse-free survival rate of the high-TILs group was significantly higher than that of the low-TILs group (p = 0.0251, Fig 4).

The five-year overall survival rate of the high-TILs group was significantly higher than that of the low-TILs group (p = 0.0059, Fig 4).

### Prognostic factors influencing the RFS

Factors with p-values of <0.1 tended to be associated with RFS. Thus, factors with p-values of <0.1 in the univariate analyses were included as covariates in a multivariate analysis.

Sex, age, lymphatic vessel invasion, lymph node metastasis, the preoperative carbohydrate antigen 19–9 (CA19-9) level and the density of TILs were significantly associated with RFS and the preoperative CEA level tended to be associated with RFS in the univariate analysis (Table 3).

**Table 3:** The results of the univariate and multivariate analyses of the prognostic factors for relapse-free survival.

	Univariate			Multivariate		
	OR	95%CI	p-value	OR	95%CI	p-value
Sex (male)	1.881	1.029–3.561	0.0442	2.223	1.176–4.345	0.0137
Age (≥67years)	2.201	1.187–4.286	0.0118	2.556	1.345–5.079	0.0039
Tumor location (Rectum)	1.115	0.614–2.024	0.7186			
Tumor size (≥4cm)	0.962	0.530–1.746	0.8989			
Depth of tumor (T4)	1.030	0.529–1.910	0.9275			
Lymphatic vessel invasion (positive)	2.362	1.074–6.233	0.0313	1.219	0.506–3.404	0.6734
Venous invasion (positive)	1.721	0.851–3.256	0.1256			
Lymph node metastasis (positive)	2.389	1.313–4.472	0.0043	1.944	0.989–3.930	0.0539
Preoperative CEA level (≥5ng/ml)	1.740	0.951–3.150	0.0718	0.774	0.357–1.588	0.4918
Preoperative CA19-9 level (≥ 37U/ml)	3.837	1.826–7.505	0.0008	3.967	1.667–9.137	0.0023
The density of TILs (High)	2.585	1.177–6.813	0.0160	2.342	1.031–6.311	0.0414

OR, odds ratio; CI, confidence interval; CEA, carcinoembryonic antigen; CA19-9, carbohydrate antigen 19–9; TILs, tumor infiltrating lymphocytes

The multivariate analysis showed that sex (odds ratio [OR], 2.223; 95% confidential interval [CI], 1.176–4.345; p = 0.0137), age (OR, 2.556; 95%CI, 1.345–5.079; p = 0.0039), preoperative CA19-9 level (OR, 3.967; 95%CI, 1.667–9.137; p = 0.0023) and the density of TILs (OR, 2.342; 95%CI, 1.031–6.311; p = 0.0414) were independently associated with the RFS (Table 3).

### The prognostic factors influencing the OS

Factors with p-values of p<0.1 tended to be associated with OS. Thus, factors with p-values of <0.1 in the univariate analyses were included as covariates in a multivariate analysis. Age, venous invasion and the density of TILs were significantly associated with OS in the univariate analyses (Table 4).

**Table 4:** The results of the univariate and multivariate analyses of the prognostic factors for overall survival.

	Univariate			Multivariate		
	OR	95%CI	p-value	OR	95%CI	p-value
Sex (male)	1.575	0.724–3.595	0.2537			
Age (≥67 years)	3.606	1.532–9.876	0.0026	4.153	1.762–11.386	0.0008
Tumor location (Rectum)	1.141	0.524–2.486	0.7366			
Tumor size (≥4cm)	0.603	0.264–1.310	0.2029			
Depth of tumor (T4)	0.620	0.227–1.457	0.2859			
Lymphatic vessel invasion (positive)	1.134	0.482–3.105	0.7849			
Venous invasion (positive)	3.060	1.339–6.664	0.0093	2.249	0.979–4.932	0.0558
Lymph node metastasis (positive)	1.904	0.880–4.256	0.1020			
Preoperative CEA level (≥5ng/ml)	1.746	0.793–3.784	0.1626			
Preoperative CA19-9 level (≥37U/ml)	2.329	0.774–5.766	0.1223			
The density of TILs (High)	9.784	2.074–174.751	0.0012	11.617	2.453–207.707	0.0004

OR, odds ratio; CI, confidence interval; CEA, carcinoembryonic antigen; CA19-9, carbohydrate antigen 19–9; TILs, tumor infiltrating lymphocytes

The multivariate analysis showed that age (OR, 4.153; 95%CI, 1.762–11.386; p = 0.0008) and the density of TILs (OR, 11.617; 95%CI, 2.453–207.707; p = 0.0004) were independently associated with the OS (Table 4).

### The correlation between the density of TILs assessed by an observer and that assessed by an automated imaging software program

**Fig 5:**
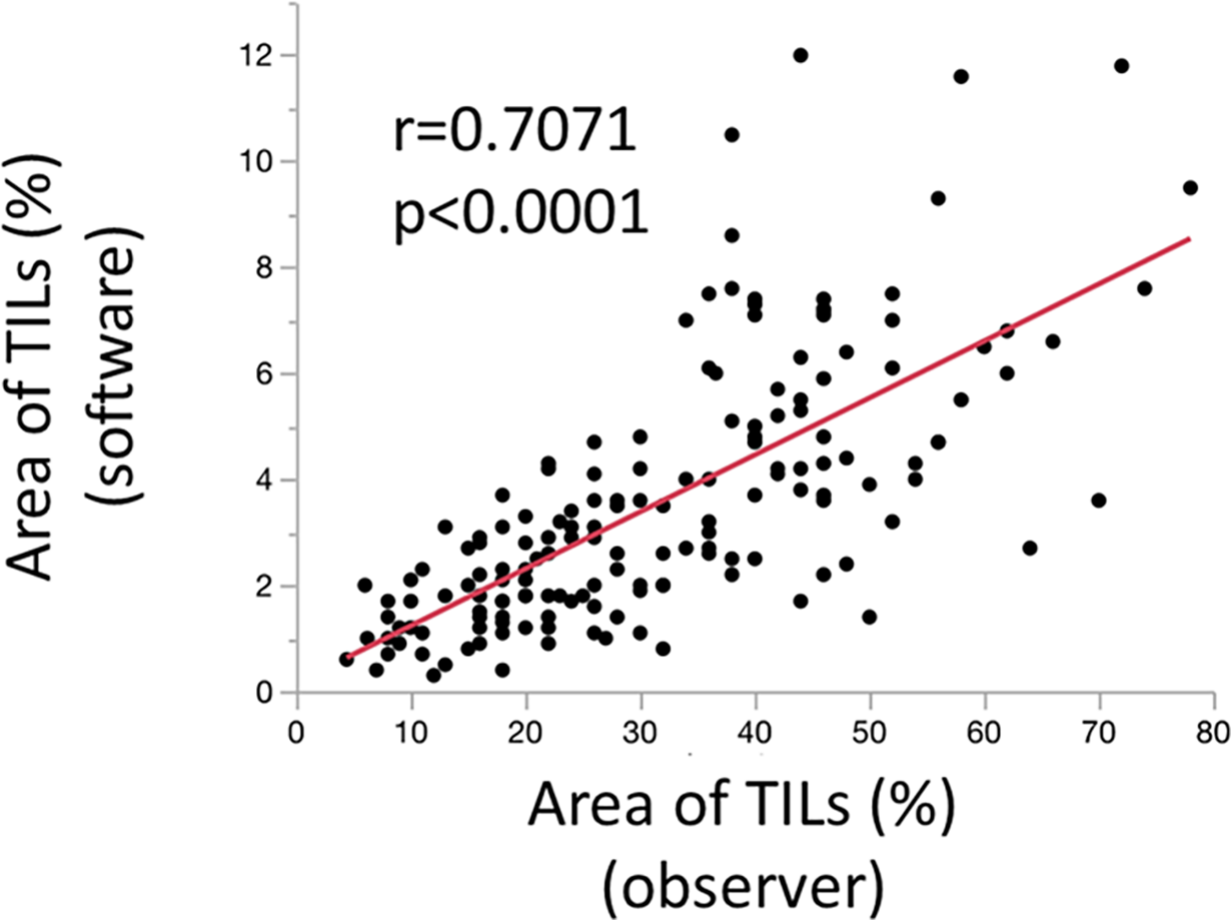
The correlation between the density of TILs assessed by an observer and that assessed by an automated imaging software program. The correlation between the density of TILs assessed by an observer and the density of TILs assessed by the imaging software program are shown. A significant positive correlation was observed (r = 0.7071, p<0.0001).

The area occupied by TILs on H-E-stained sections were also evaluated by an automated imaging software program (Fig 2). The density of TILs assessed by an observer was revealed to be significantly and positively associated with the density of TILs assessed by the imaging software program (r = 0.7071, p<0.0001) (Fig 5).

## Discussion

The extent of lymphocytic invasion in the tumor tissue reflects the immune status of the host and TILs play a pivotal role in tumor progression [4]. Some reports have shown that TILs are a good prognostic factor in CRC [3–6]. Although TILs are correlated with the prognosis in CRC, various methods have been applied to their evaluation. These include immunohistochemical or H-E staining; the evaluation of the tumor center or invasion front; and the percentage of the area occupied by TILs or the absolute number of TILs. [4–6, 8, 11–17]. Klintrup et al. [11] and Huh et al. [4] reported that a greater number of inflammatory cells was correlated with a good prognosis in CRC in a study in which TILs were assessed using a 4-degree scale. Canna et al. [5] showed that the loss of CD4+ T-lymphocytes was correlated with poor survival in a study in which immunohistochemical staining for CD4 was performed. Funada et al. [6] showed that a high level of CD8+T lymphocytes was significantly correlated with a good prognosis. Chiba et al. measured the number of TILs [18], on the other hand Klintrup et al. measured TILs by using a four-degree scale [11]. Nosho et al. measured the TILs in tumor center [14], whereas Menon et al. measured the TILs in invasive margin [19]. Although it is clear that TILs are associated with the prognosis of CRC, there is no standardized methodology for evaluating the density of TILs.

The International TILs Working Group outlined the recommendations for evaluating TILs in breast cancer in 2014 [9]. They recommended TILs be evaluated using H-E stained sections [9]. In this study, we considered the method recommended by the International TILs Working Group could be used to assess TILs in CRC as well as in breast cancer. The study indicated that the high-TIL group showed a good prognosis, in terms of RFS and OS. H-E-stained sections are easy to make; thus, the evaluation of TILs using H-E-stained sections might be useful for predicting the prognosis in CRC.

The original article by the TILs working group recommended the percentage of TILs as a continuous parameter, because they were focused on the future. They investigated the correlation between the percentage of TILs and the results of clinical trials. However, the purpose of the present study was to clarify whether or not the evaluation of TILs by an observer is useful in the clinical setting. We therefore classified the patients into two groups according to percentage of TILs, and not as a continuous parameter. Swisher et al. also suggested that the prognostic value of TILs is a non-continuous variable rather than a continuous variable [20]. We treated the five-year relapse-free survival as the state variable and the percentage of tumor infiltrating lymphocytes (TILs) as the test variable in the present study. A ROC curve analysis revealed that the most appropriate cut-off TIL percentage was 42%.

We considered the objectivity of the evaluation of TILs by an observer might not be sufficiently demonstrated. Swisher et al. assessed the reproducibility of the observers’ measurements and the interobserver agreement of TIL assessments, which was advocated by the TILs working group [20]. In this report, they observed acceptable agreement in the TILs working group’s method of evaluating TILs [20]. Thus, in the present study, only one observer counted the TILs; however, we considered that this secured the quality of the results. Moreover, we used an automated imaging software program to confirm the validity and objectivity of the evaluation of TILs by an observer. The density of TILs determined by an observer was found to be correlated with the density determined using the automated imaging software program. Thus, we confirmed the validity of the evaluation of TILs by an observer. Although an automated imaging software program is tended to be recognized to be objective and superior to observers, the evaluation of TILs using automated imaging software programs is associated with some problems. Teng et al. suggested that the evaluation of TILs by observers was superior to the evaluation of TILs using an automated software program, because automated imaging software programs distinguished cells based on a different depth of color without considering the shape, features, or positions [12]. Although evaluations using automated imaging software programs are objective, it was hard to limit the selection of cells to those that we wanted to evaluate in the present study. The method for limiting cell selection is a problem that remains to be solved. Moreover, the accuracy of the area of TILs that was determined by the software was affected by the fact that the software only distinguishes cells based on the different depth of color. If we expand the range of the depth of color, the area of TILs would increase; however, the density of TILs may be overestimated because of cells other than inflammatory cells (which should not be evaluated) are selected. Conversely, if we narrow the range of the depth of color, the area of TILs would be underestimated, because heterogeneity of shade exists even in one lymphocyte. Further examination is necessary to determine the appropriate settings, and the differences between the software. We believe that evaluation by observers is currently more useful for evaluating the density of TILs than the method by the automated imaging software program.

The heterogeneity in a single tumor section remains a problem. The International TILs Working Group recommended that different regions should be evaluated and that the average density of TILs should be reported [9]. In the present study, we assessed five independent microscopic fields for each patient sample and reported the average.

The present study was associated with some limitations. First, this study was a retrospective, single-center study with a relatively small number of patients. Further studies, including prospective studies with a larger number of patients, should be performed to confirm our findings. Secondly, we did not assess the subtype of TILs. CD3+, CD8+, CD4+, CD45+, FOXP3+ TILs have been reported to be associated with favorable survival. [5, 12–14, 16]. Although there have been numerous reports on the correlation between subtypes of TILs and the prognosis of CRC, we did not assess the clinical importance of subtyping TILs in this study. However, the International TILs Working Group does not currently recommend that immunohistochemistry be used to detect specific subpopulations, and recommended that the measurements on H-E-stained sections should be used to evaluate TILs at the present time. In the near future, we would like to clarify the association between the evaluation of TIL subpopulations and the evaluation of TILs using H-E-stained sections. Thirdly, it remains unclear as to whether the leading edge of the tumor is really the optimal site for evaluating the immune status of the host or not. Kim et al. reported that the invasive front of the tumor most accurately reflects the character of cancer in patients with CRC; thus, the invasive front is considered to be the optimal site for evaluating TILs [21]. Based on their approach, we evaluated the TILs at the invasive front of tumor. Finally, the TILs that were evaluated in this study were mononuclear cells, including lymphocytes, plasma cells and other inflammatory cells. With regard to these points, the International TILs Working Group recommended the scoring of all mononuclear cells, including lymphocytes and plasma cells (granulocytes and other polymorphonuclear leukocytes were excluded); however, the quantitative assessment of other mononuclear cells such as dendritic cells and macrophages is not currently recommended [9]. Thus, we evaluated all nuclear cells as TILs in the present study. However, it has been reported that they affect the prognosis of CRC; thus, further studies should be performed to confirm our findings.

### Conclusion

In conclusion, this study suggested that the evaluation of TILs using H-E-stained sections is easy and that the results might be used to predict the prognosis of CRC. Moreover, the results obtained in the evaluation of TILs on H-E-stained sections by an observer were positively correlated with the results obtained using an automated imaging software program. Thus, the evaluation of TILs by an observer may provide objective results.
